# Quantitative Assessment of Secondary Healthcare Utilisation by Patients With Functional Abdominal Pain

**DOI:** 10.7759/cureus.25145

**Published:** 2022-05-19

**Authors:** Emma MacVicar, Pauline Insch, Fiona Summers, Duff Bruce, George Ramsay

**Affiliations:** 1 Medicine and Surgery, University of Aberdeen School of Medicine, Aberdeen, GBR; 2 Clinical Psychology, NHS Grampian, Aberdeen, GBR; 3 Colorectal Surgery, NHS Grampian, Aberdeen, GBR; 4 Health Services Research Unit, University of Aberdeen, Aberdeen, GBR

**Keywords:** healthcare costs, general surgery, functional bowel disorder, clinical audit, functional abdominal pain syndrome

## Abstract

There is increasing awareness of the impact functional conditions have on the National Health Service (NHS). Less is known about the resources used to manage these conditions. This retrospective quantitative audit aims to determine the demographic and healthcare utilisation of functional abdominal pain patients presenting to the hospital.

The most frequent hospital attenders with non-specific abdominal pain in NHS Grampian, 2018-2019, were assessed (n=144). Adult patients meeting the ROME II diagnostic criteria for functional abdominal pain diagnosis were included (n=33). Data were retrospectively collected manually from electronic medical records.

Of 33 patients, 93.9% were female, with a mean age of 31.2 years. Each had accessed a mean of 11.5 specialist services, with 69.7% being referred to mental health services; 9.1% had completed treatment. Each patient had a median 4 (range 1-26) emergency/unscheduled presentations to hospital and median 2 (range 0-13) admissions for functional abdominal pain during the study period, with a total of 247 nights spent in hospital by this patient cohort for functional abdominal pain alone. The estimated total cost for these hospital admissions was £593,786.00.

Extensive secondary-care input is currently required for patients with functional abdominal pain at a significant cost. Patients are re-presenting to the hospital frequently, which suggests that current management is not effective.

## Introduction

In recent years, there has been an increased focus on, education, and awareness of functional disorders. The significant impact these pathologies have on patients, staff, and health services is also better appreciated. Functional disorders are present in all areas of medicine and pose major challenges for all specialities [1]. Functional abdominal pain is defined as continuous abdominal pain with no known underlying biochemical or structural pathology to explain the symptoms; specific diagnostic criteria are detailed below [2]. Patients with functional abdominal pain may present complaining of dyspepsia, non-specific or shifting abdominal pain, nausea or other gastrointestinal signs. The estimated prevalence of these presentations in North America was estimated to be 0.5-1.7% in 1993, with young women, in particular, more likely to report functional abdominal pain than males [3]. No recent epidemiological studies have been carried out in the United Kingdom (UK) as far as the authors are aware.

There appears to be a complex, multifactorial aetiology for functional abdominal pain involving abnormal brain-gut pain regulation (e.g. magnified ascending signals or impaired descending inhibitory pathways) [4-7]. There is also a clear association in both children and adults between functional abdominal pain and psychiatric/psychological conditions [8-10]. A recent meta-analysis showed a significant association between a history of sexual abuse and diagnosis of functional gastrointestinal disorders [11], and these patients have also been found to report more severe forms of abuse compared to those with structural pathology [9].

Five diagnostic criteria for functional abdominal pain syndrome have been defined as part of the ROME II multi-consensus committee. These include continuous or near-continuous abdominal pain, some loss of daily functioning, pain not feigned, no or only occasional relation to physiological events, and do not fit the criteria for other diagnoses. In clinical practice, the diagnosis is based on history and clinical examination, the absence of ‘red-flag’ or alarm symptoms (which may suggest an organic cause but do not completely rule out a functional disorder) [12] and negative investigations [5,13-14].

The overall goal of treatment is to help patients manage and cope with their chronic pain symptoms rather than aiming for a cure [6]. Treatment recommendations are largely empirical and not based on good-quality, well-designed clinical trials [2]. A variety of treatments have been used to help patients, including cognitive behavioural therapy (CBT), hypnosis, relaxation techniques, and anti-convulsant and anti-depressant medications [5-6,15]. The importance of a ‘therapeutic patient-physician relationship’ is also highlighted in the literature [2,16]. There is therefore a need for clinicians to listen to patients, show empathy and schedule regular routine follow-up appointments so patients are not left to present to emergency services in distress [6].

It is recognised that this patient group have particularly high healthcare utilisation [3,6]. However, precise quantification of the healthcare usage of these patients remains unclear. This study aims to quantify the current secondary care input required for this group of patients in a single, tertiary UK hospital.

## Materials and methods

Study design

This is a single-centre, retrospective cohort study.

Patient selection

Aberdeen Royal Infirmary (ARI) is a tertiary care hospital in the North-East of Scotland, UK, managing the healthcare requirements of a population of approximately 580,000 individuals. This is the first time an audit of this topic has been conducted at ARI and there are limited data available in the literature, therefore a two-year time period was chosen for this initial pilot investigative study.

The National Health Service (NHS) Grampian Health Intelligence Team collated the Community Healthcare Index (CHI) numbers and demographic data for all patients discharged from hospital with a non-specific abdominal pain (NSAP) diagnosis in NHS Grampian over a two-year period: January 1, 2018, to December 31, 2019.

Adult patients (aged 18 years or older) with a functional abdominal pain diagnosis confirmed by their secondary-care team (meeting the ROME II diagnostic criteria for the diagnosis of functional abdominal pain) and attending hospital most frequently with symptoms (3 or more NSAP discharges coded in their record) were included in this audit. Those with a diagnosis of organic pathology or individuals attending less frequently to hospital were excluded.

Data collection

The Health Intelligence Team of NHS Grampian provided age, sex, post-code, and geographical locality for each patient. Postcode data were then used to calculate the Scottish Index of Multiple Deprivation (SIMD) quintile score for each patient using the Scottish Government website, 2020 [17]. SIMD is an area-based measure of deprivation, used by the Scottish Government to identify areas of deprivation, i.e., those with low incomes or fewer resources/ opportunities.

Community Health Index (CHI) numbers were used to access electronic medical records to allow co-morbidity and health care utilisation data to be collected by searching the medical records manually. Data were stored securely on the NHS Grampian server.

The mean number of outpatient appointments (i.e., scheduled presentations to the hospital) for all specialities (2018-2019) was collated: cancelled, rescheduled or did-not-attend (DNA) appointments were not included. Unscheduled (emergency) hospital presentations for abdominal pain with same-day discharge from hospital and hospital admissions data (number of admissions and total number of nights spent in hospital related to functional abdominal pain 2018-2019) are summarised. Data on imaging conducted during 2018-2019, including the number of radiographs, computerised tomography (CT), magnetic resonance imaging (MRI), ultrasound scans, and endoscopic examinations were also collected manually from the electronic records. Longer-term imaging data from 2011 to 2019 (from the introduction of electronic medical records in NHS Grampian) are also included.

Ethical approval

This study was registered as an audit with Clinical Effectiveness - NHS Grampian. No further ethical approvals were deemed necessary.

Analysis of data

Descriptive statistics to summarise the patient demographic and healthcare utilisation are used throughout. Data were stored in Microsoft Excel (Microsoft Corporation, Redmond, WA). The mean and standard deviation (SD) for patient demographic data, imaging modalities, co-morbid conditions and number of specialities referred to per patient was calculated: where the distribution of data is skewed median and range are quoted. Mean, SD and percentages are rounded to one decimal place.

Estimated costs of treatment were calculated using values taken from Public Health Scotland [18] except for the estimated cost of an endoscopy exam, which was approximated at £372.00 [19]. Radiography exposure was calculated using values taken from the UK government ‘Patient Radiation Dose Information Guidance’. The average millisievert (mSV) dose per CT scan was calculated at 6.67 mSV and the average radiation dose per radiograph was 0.46 mSV [20].

## Results

The patient demographic is summarised in Table 1. Patients were primarily female (93.9%) with a mean age of 31.2 years (SD 13.6, age range 18-77 years). Average body mass index (BMI) was 31.4 kg/m^2^ (moderately obese category) and ranged from 20.7-50.4 kg/m^2^. Patients were most likely to live in deprived areas with 72.7% of patients residing in the three most deprived SIMD categories (quintiles 1, 2 and 3). Fifteen (45.5%) were current or ex-smokers. The majority of this patient cohort were in employment or registered as a student (60.6%), with 24.2% unemployed or on long-term sick leave.

**Table 1:** Summary of Patient Demographics SIMD: Scottish Index of Multiple Deprivation

	Audited Patients (n=33)
Age	(years)
Range (min-max)	18-77
Mean	31.2 (SD 13.8)
Gender	Number (Percentage)
Male	2 (6.1%)
Female	31 (93.9%)
SIMD Quintile	Number (Percentage)
SIMD Areas 1+2 (most deprived)	19 (57.6%)
SIMD Area 3	5 (15.2%)
SIMD Areas 4+5 (least deprived)	9 (27.3%)
Employment Status	Number (Percentage)
Employed/ Student	20 (60.6%)
Unemployed/ Long-Term Sick Leave	8 (24.2%)
No Record	5 (15.2%)

Patients had a minimum of two co-morbid conditions and a maximum of 11. In total, there were 208 diagnoses recorded for this patient population with a mean of 6.3 (SD 2.6) per patient. There were 55 diagnoses in total with an organic cause, incorporating a wide range of conditions with little overlap between patients. Examples include asthma, angina and osteoarthritis.

Thirty patients (90.9%) had more than one functional diagnosis, with eight (24.2%) diagnosed with four or more functional conditions; examples include irritable bowel syndrome, non-epileptic seizures, functional muscle weakness and fibromyalgia. The categories of co-morbid conditions are summarised in Table 2.

**Table 2:** Categorisation of Patient Co-Morbidities

Category	Total Number	Range Per Patient	Mean (SD)
Psychological/Psychiatric	65	0 - 7	2.0 (SD 1.9)
Functional	88	1 - 5	2.7 (SD 1.0)
Organic	55	0 - 5	1.7 (SD 1.5)
TOTAL	208	2 - 11	6.3 (SD 2.6)

The most frequent co-morbid conditions were irritable bowel syndrome (51.5%), anxiety (45.5%), depression (39.4%), and self-harm, including attempted suicide, deliberate self-injury, and drug overdose (33.3%). Twenty-six (78.8%) had at least one psychiatric or psychological diagnosis recorded, with 22 patients (66.7%) diagnosed with depression and/or anxiety. More than half of this patient cohort (51.5%) had a noted emotional/psychological and, in some cases, physical/sexual trauma history; examples include domestic abuse, childhood sexual abuse, and gang attacks. Six (18.2%) reported no history of psychological/physical trauma and 10 (30.3%) had no record on their electronic medical record of any discussion regarding trauma; no objective measure was used to measure trauma for any patient and there was no evidence of standardised questioning.

Engagement with mental health services was further assessed. Twenty-three (69.7%) patients had been referred to psychiatry or psychology services. Three (9.1%) had completed a course of treatment. Nine (27.3%) were actively engaged in treatment or on the waiting list at the end of the study period.

Patients were referred to a total of forty-five individual specialities, ranging from four to 19 specialities per patient. The mean total per person was 11.5 (SD 3.9). The three most common specialities referred to were general surgery (90.9%), gastroenterology (84.8%) and anaesthetics (78.8%). Almost a third of patients were not referred to mental health services: only 69.7% were referred to psychiatry or psychology. Thirteen (39.4%) patients were referred for dietetic support and 13 (39.4%) were referred to the pain management team. Only one (3.0%) patient required nasogastric feeding (due to significant psychological comorbidity resulting in low intakes and weight loss); the majority (97.0%) did not require supportive feeding by tube-feeding or total parenteral nutrition. For all specialities, patients had an average total of 19 (SD 18.6) outpatient appointments (2018-2019) ranging from one to 73 per person. The total calculated cost of these outpatient visits was estimated at £115,996.00 over the two-year period.

Imaging for each patient from 2018 to 2019 was also analysed. Patients had a median of two (range 0-14) radiographs, two (range 0-10) ultrasound scans, one (range 0-4) CT scan, one (range 0-5) MRI scan and three (range 0-3) endoscopic investigations during this time period. There was marked variation between patients with regards to the number of investigations carried out. The total number of imaging investigations for these 33 patients was 348. Patients in this cohort received a median 6.67 mSV radiation dose from CT scans alone in 2018-2019. The most frequently scanned patient (four CT scans) received 26.68 mSV over this time period. In addition, these patients were exposed to a median of 0.92 mSV radiation from radiographs; the most frequently radiographed patient (14 radiographs) had an additional dose of radiation equal to 6.44 mSV. The total estimated costs for imaging investigations for this cohort of 33 patients (2018-2019) was £39,798.64. The mean cost was calculated at £1206.02 per head (Table 3).

**Table 3:** Summary of Imaging and Estimated Financial Cost for 33 Audited Patients (2018-19)

Imaging Modality	Number of Patients Imaged	Percentage of Audit Cases Imaged	Total Number	Median Number	Range	Approximate Unit Cost	Estimated Total Cost	Mean Cost (/33 total patient cohort)
Ultrasound	29	87.90%	124	3	0-10	£58.20	£7,216.80	£218.69
Radiograph	26	78.80%	117	2	0-14	£77.49	£9,066.33	£274.74
CT	24	72.70%	43	1	0-4	£116.27	£4,999.61	£151.50
MRI	20	60.60%	34	1	0-5	£216.35	£7,355.90	£222.91
Endoscopy	21	63.6%	30	3	0-3	£372.00	£11,160.00	£338.18
TOTAL			348				£39,798.64	£1,206.02

In addition, the total data available since the TrakCare electronic record system (InterSystems Corporation, Cambridge, Massachusetts) was introduced in NHS Grampian (2011-2019) was analysed to give more detail regarding the total imaging required for this cohort of patients over time and showed that during this time, 97.0% of this patient cohort had at least one radiograph. Patients had a median of seven (range 0-86) radiographs, five (range 0-25) ultrasound scans, two (range 0-10) CT scans, one (range 0-6) MRI scan, and one (0-6) endoscopic investigation. The total number of imaging investigations for these 33 patients, 2011-2019, was 803, highlighting the extensive and long-term secondary care input required for these patients.

Secondary care contacts and estimated costs over the two-year period are summarised in Table 4. Each patient had median 4 (range 1-26) emergency or unscheduled presentations to the hospital in relation to abdominal pain and gastrointestinal symptoms. Outcomes of these presentations were a median of 2 (range 0-13) same-day-discharge appointments and a median of 2 (range 0-13) admissions to hospital over the two-year period. In total, 247 nights were spent in the hospital for functional abdominal pain by this patient cohort: a median of four (range 0-34) nights per patient. There was wide variation between patients in the total number of nights spent in the hospital related to the overall number of admissions required; for example, one patient was admitted for functional abdominal pain 13 times during the two-year audit period. Hospital admission and emergency presentation costs are estimated at £477,790 in total, a mean of £14,478.48 for each person audited.

**Table 4:** Summary of Unscheduled Appointments and Admissions for Functional Abdominal Pain Only Over a Two-Year Period (2018-2019) for 33 Audited Patients

Type of Contact	Number of patients	Percentage Patients	Total	Median	Range	Unit Cost/Case	Estimated Total Cost	Mean Cost Per Patient
Same-Day Discharge Appointments	25	75.8%	69	2	0-13	£1046.00	£72,174.00	£2187.09
Number of Hospital Admissions	30	90.9%	101	2	0-13	£4016.00	£405,616.00	£12,291.39
TOTAL COSTS							£477,790.00	£14,478.48

## Discussion

This retrospective quantitative study found that patients with functional abdominal pain attending the hospital most frequently were of a similar demographic and repeatedly attend secondary care despite extensive investigation, hospital admissions and explanation that the underlying cause of their pain is functional. This suggests that our current methods of managing this condition are ineffective.

This group of young, mainly female, patients was found to have multiple co-morbid conditions. There was a particularly high prevalence of psychiatric/psychological disorders and functional disorders, in keeping with previous research into functional abdominal pain [9]. They also had a high number of referrals (mean 11.5 per person) to different specialities, resulting in many different professionals being involved in patient care. It is difficult to see how any continuity of care can exist and the current system for managing these complex patients does not allow any single clinician to have a clear overview of the diagnosis and full history. Furthermore, this appears to demonstrate a preponderance of the clinical specialities concentrating on ruling out physical conditions rather than managing the functional presentations within this cohort of individuals.

The most referred to speciality was General Surgery (90.9% of the cohort). Although general surgeons are experts in diagnosing and managing surgical causes of abdominal pain, are they the best specialists to manage these patients, particularly once they have a functional abdominal pain diagnosis? A general surgeon’s knowledge and experience of the most up-to-date psychological treatments or anti-depressant medications for functional disorders may well be limited. Considering that such a large percentage of these patients are presenting to general surgery, further training and development for surgeons in the presentation and management of functional abdominal pain and other functional conditions would be beneficial. A multi-disciplinary team approach to treatment across specialities is likely to be required as these patients are being seen, investigated and treated by many different services. The underlying difficulty for healthcare staff assessing these patients is the fear of missing a treatable or potentially life-altering organic or biochemical diagnosis, particularly cancer [5]. The high number of investigations found in this audit may well reflect this underlying concern. In addition, patients with functional abdominal pain have been shown to exhibit certain behaviours that likely contribute to the level of hospitalisations and investigations conducted.

Burton et al., in 2020, proposed that all functional disorders should be grouped in one class - suggesting the name ‘Functional Somatic Disorders’ (FSD) - because of the similar aetiology and overlap between patients presenting with functional symptoms [21]. Our findings agree with this observation that there is significant overlap between different functional conditions. It, therefore, follows that managing patients as a whole person by adopting a more complete, holistic approach rather than splitting them into individual specialities or diagnoses would perhaps be a more effective way to help these patients, reduce unnecessary interventions, and, as another advantage, reduce the financial cost to the NHS.

We also note a wide degree of variation between patients in the number and type of investigations performed. There does not appear to be a clear endpoint or plan for patients presenting repeatedly with functional abdominal pain in our unit at this time. One excellent example of a functional abdominal pain guideline that could be modelled is in Brighton and Sussex University Hospitals for paediatric patients [15]. This emphasises the importance of looking at biological, psychological and social factors in the management of symptoms and gives helpful information for the clinician regarding how to discuss functional disorders with patients and families and explain the diagnosis. Developing a clear guideline and including criteria for reassessment and evaluation for red-flag signs is important to increase clinician confidence and improve patient care.

It is interesting that despite extensive support at outpatient clinics and a clear functional diagnosis for their abdominal pain, patients continued to present as unscheduled emergency cases with abdominal pain and require admissions to a hospital. This suggests that their symptoms were not well-controlled and that the current management of their condition is ineffective.

Exposure to radiation from repeated radiological tests is a significant public health concern due to the risks of causing solid tumours and haematological malignancies [22]. Women (93.9% of patients in this study population) have been shown to have a higher risk of damage from radiation exposure than men. The highest risk is seen with repeated radiation exposure, particularly at a young age [22]. The risk of radiation inducing cancer is estimated to be 0.5% at a dose of 100 mSV - the most irradiated patient in the study by age 27 had received an estimated 106.26 mSV total dose [23]. The median estimated radiation dose received by each patient in this study was 7.59 mSV radiation, 2018-2019. Thus, not only are the current methods of managing these patients insufficient in terms of readmission rates and reattendances, the investigations undertaken during this management have the potential to cause longer-term morbidity, in particular for patients who are undergoing the highest number of imaging examinations.

We acknowledge the following limitations: this was a retrospective quantitative study and data were stored in the electronic medical records. This work, therefore, relies on the accuracy of the data and there is a risk of human error in recording. This study looks at a very small group of patients (n=33), who are patients diagnosed with functional abdominal pain who attended hospital with symptoms from 2018 to 2019. Although these are an important patient group to assess, with particularly high healthcare utilisation, they do not represent the patient population as a whole. It was also not possible to separate the imaging data into that conducted for abdominal pain vs for other conditions. Instead, we have an overview of the total imaging for each patient. The same is true for outpatient appointments; these do not relate specifically to functional abdominal pain and, instead, give an overview of hospital outpatient clinic visits for all specialities. Costs of treatment are estimated from the data available: the true cost is likely to be much higher, particularly when primary care data is taken into account.

## Conclusions

This audit is the first time the demographic and health care utilisation of patients presenting to secondary care with functional abdominal pain in NHS Grampian have been analysed. The audit has shown that this patient group is of a similar demographic and is repeatedly attending hospital - both as outpatients and as emergency presentations/admissions - and undergoing extensive investigations. This results in repeated radiation exposure, high financial costs and frequent interventions with potential for harm (both psychological and physical), and it seems of little benefit to these patients. 

NHS Grampian needs to develop more effective ways to support and help this patient group who have particularly high health-care utilisation. A specialist patient-centred approach is likely to be most effective, working alongside patients to understand what support they would find helpful. It is important to determine methods of increasing patient engagement with mental health services, and develop a more holistic treatment approach, preventing unnecessary investigations, hospital stays and the associated risks for patients. A clear, defined diagnosis and treatment pathway for functional abdominal pain, which includes indications for further investigation/review (e.g., if the patient develops ‘red-flag’ symptoms) should be developed in NHS Grampian.
